# First report of *Xiphinema hunaniense* Wang & Wu, 1992 (Nematoda: Longidoridae) in Vietnam

**DOI:** 10.21307/jofnem-2020-078

**Published:** 2020-07-28

**Authors:** Huu Tien Nguyen, Thi Duyen Nguyen, Thi Mai Linh Le, Quang Phap Trinh

**Affiliations:** 1 Institute of Ecology and Biological Resources, Vietnam Academy of Sciences and Technology, 18 Hoang Quoc Viet, Cau Giay, 100000, Hanoi, Vietnam; 2 Graduate University of Science and Technology, Vietnam Academy of Sciences and Technology, 18 Hoang Quoc Viet, Cau Giay, 100000, Hanoi, Vietnam; 3 Nematology Research Unit, Department of Biology, Ghent University, K.L. Ledeganckstraat 35, 9000, Ghent, Belgium

**Keywords:** 28S rDNA, Dagger nematode, Molecular identification, Plant-parasitic nematodes, Taxonomy, *Xiphinema hunaniense*

## Abstract

For the first time, a survey of plant-parasitic nematodes in the Central Highlands of Vietnam discovered a population of *Xiphinema hunaniense* Wang & Wu, 1992. The Vietnamese population of *X. hunaniense* is characterized by having an offset lip region, lack of anterior genital branch, vagina directed backward, and a digitate tail. Morphological features and morphometrics of this population are in agreement with the type population of *X. hunaniense* except for some variations. In addition, molecular characterization of this population and phylogenetic tree of 28S rDNA sequences of the genus are also provided.

The genus *Xiphinema* Cobb, 1913, commonly known as dagger nematodes, are migratory ectoparasitic nematodes that damage numerous wild and cultivated plants through direct feeding on the root and transmission of plant viruses (Taylor and Brown, 1997; Perry and Moens, 2013). This genus is distributed worldwide and is divided in two groups, *Xiphinema americanum* group and non-*Xiphinema americanum* group, with more than 260 valid species (Gutiérrez-Gutiérrez et al., 2012). The conserved morphology and overlapping morphometrics of some species groups in the genus *Xiphinema* make quarantine regulations and protection methods more difficult. Therefore, accurate identification of *Xiphinema* species using integrate approach is strongly recommended to create a basis for plant pest management.

In Vietnam, eight species of the genus *Xiphinema* have been reported, however, molecular identification are not available for most of them (Nguyen and Nguyen, 2000), and thus, a higher diversity of *Xiphinema* spp. is expected in the country with the use of molecular tools. Herein, a population of *Xiphinema hunaniense* Wang & Wu, 1992 in Vietnam is characterized by the combination of morphological characters and molecular data.

## Material and methods

Soil and root samples were collected from the upper 30 cm layer of forest soil in the Central Highlands of Vietnam. Nematodes were extracted and permanent slides were made following Nguyen et al. (2019a). Pictures and measurements were recorded using Carl Zeiss Axio Lab. A1 light microscope equipped with a Zeiss Axiocam ERc5s digital camera. For molecular characterization, the 5′-end region of 28S rDNA was amplified using DP391/501 primers (5′-AGCGGAGGAAAAGAAACTAA-3′/5′-TCGGAAGGAACCAGCTACTA-3′) following Nguyen et al. (2019b). Forward and reverse sequences were assembled and analyzed using Geneious R11 (Nguyen et al., 2019b, 2019c). The best fit model was chosen using Mega 7 following Nguyen et al. (2019b).

## Results and discussion

### Measurements

Eight females: L = 2,095 ± 130 (1,947-2,189) µm, V% = 24.7 ± 0.4 (24.2-25), Odontostyle = 126 ± 5 (120-130) µm, Odontophore = 71 ± 1 (70-71) µm, Stylet = 196 ± 6 (190-201) µm, Tail length = 45 ± 1 (44-46) µm, Lip width = 13.6 ± 0.1 (13.5-13.7) µm, Lip height = 5.4 ± 0.2 (5.2-5.6) µm, Pharynx = 359 ± 3 (356-361) µm, Anterior end to guiding ring = 121 ± 7 (114-127) µm, Width at pharyngo-intestinal junction = 44 ± 1 (43-44) µm, Width at mid-body = 46 ± 1 (45-47) µm, Width at anus = 29 ± 2 (27-31) µm, *a* = 46 ± 2 (43-48), *b* = 5.8 ± 0.4 (5.4-6.1), *c* = 46 ± 4 (42-49), *c*′ = 1.6 ± 0.1 (1.5-1.7).

### Remarks

**Figure 1:**
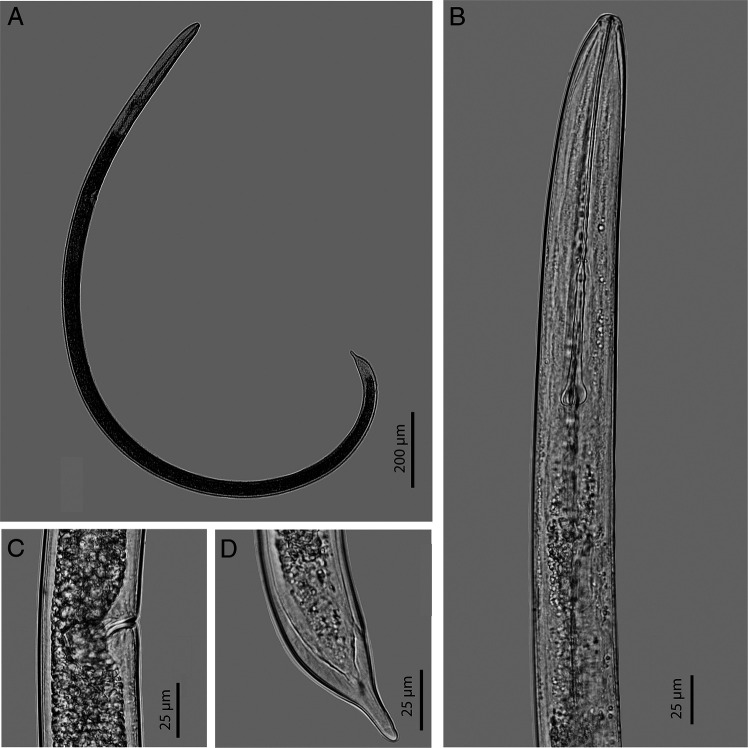
*Xiphinema hunaniense* from Vietnam. A: entire body; B: Pharyngeal region; C: Vulva region; D: Tail region.

**Figure 2:**
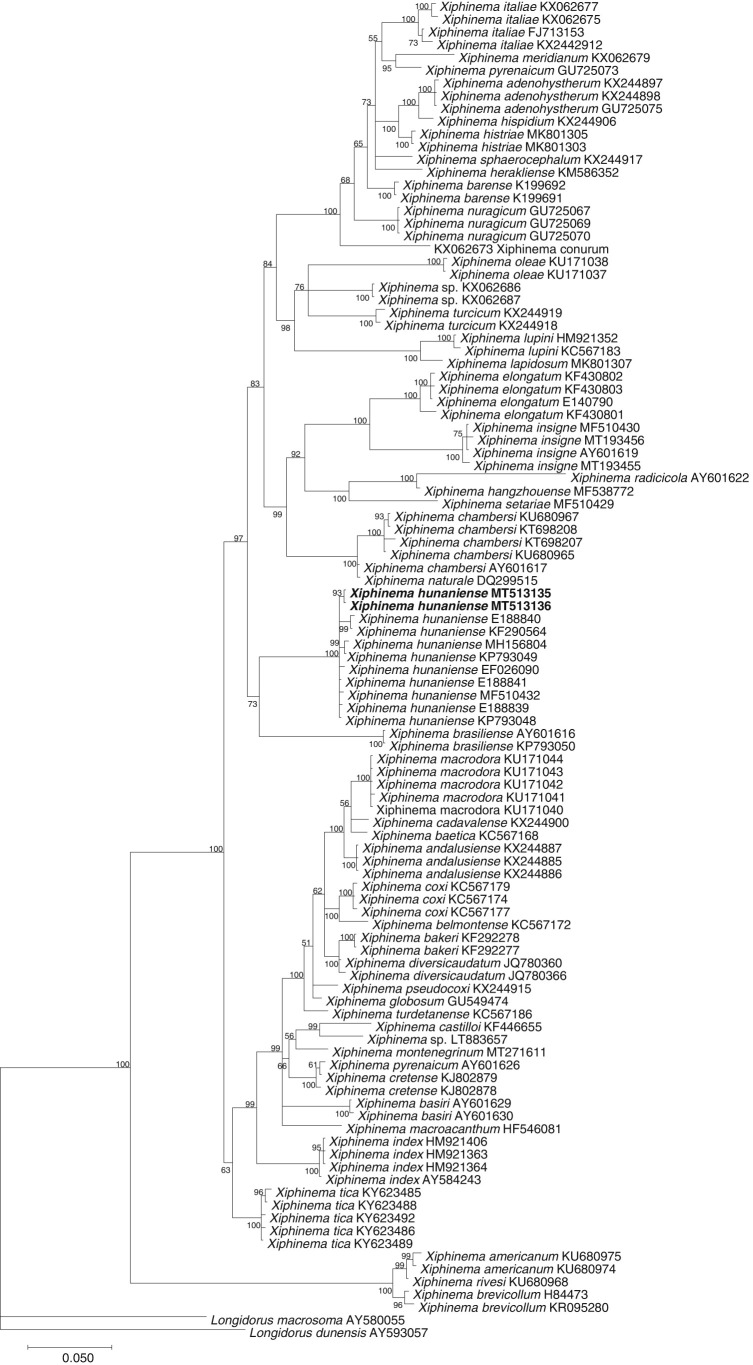
BI phylogenetic tree generated from 28S rDNA sequences (GTR + G + I model, 1 × 10^6^ generations, 20% Burn-in). Bayesian posterior probabilities (in percentage) are given next to each node. Sequences of *X. hunaniense* from Vietnam are in bold font.

The females of the Vietnamese population of *X. hunaniense* is characterized by an offset lip region from body contour, lack of anterior genital branch, vagina directed slightly backward, and a digitate tail (Fig. 1). Morphology and morphometrics of this population are highly similar to the type population of *X. hunaniense* except for smaller *a* value (43-48 vs 51-57), *c* value (42-49 vs 53-63), longer stylet (190-201 µm vs 180-187 µm), wider width at pharyngo-intestinal junction (43-44 µm vs 21-23 µm). However, these morphometric variations have been reported from other populations of *X. hunaniense* (Luc, 1981; Wu et al., 2007; Long et al., 2014). Two 28S rDNA sequences (1 bp difference) of the Vietnamese population of *X. hunaniense* were obtained, 942 to 944 bp long. These sequences are 98.9 to 99.5% similar (3-8 bp difference) to 28S rDNA sequences of *X. hunaniense* from other populations. The Bayesian inference phylogenetic tree showed that 28S rDNA sequences of the Vietnamese population of *X. hunaniense* were placed together with sequences of *X. hunaniense* from other populations (100% PP) and this group has a sister relationship (73% PP) to the sequences of *X. brasiliense* (Fig. 2).

When it comes to morphology, *X. hunaniense* is closest to *X. radicicola*, and therefore, it has been synonymized with *X. radicicola* by Loof et al. (1996). However, based on the observation of different populations of *X. hunaniense* and *X. radicicola*, Robbins and Wang (1998) re-established *X. hunaniense* as a valid species that was agreed by Zheng and Brown (1999). 28S rDNA sequences of *X. hunaniense* has a sister relationship to *X. brasiliense*, but *X. hunaniense* can be differentiated from *X. brasiliense* by the moderately offset terminal peg vs distinct peg-shaped tail and *X. brasiliense* usually has more posterior vulva position. Besides, the 28S rDNA sequences of *X. hunaniense* from Vietnam were only 87 to 88% similar (84-85 bp difference) to *X. brasiliense*.

Due to the conserved morphology in some *Xiphinema* species groups, i.e. *X. hunaniense* – *X. radicicola* – *X. brasiliense* (Zheng and Brown, 1999) or *X. americanum* group (Gutiérrez-Gutiérrez et al., 2012), the combination of morphological characters and molecular data is needed to identify *Xiphinema* species. This is the first report of *X. hunaniense* in Vietnam with the support of molecular data of 28S rDNA sequences, adding to the total number of nine *Xiphinema* species in Vietnam, including *X. americanum*, *X. brasiliense*, *X. brevicolle*, *X. diffusum*, *X. elongatum*, *X. insigne*, *X. longicaudatum*, *X. radicicola*, and *X. hunanie* *nse*.
